# Supplementary material for: Content-rich biological network constructed by mining PubMed abstracts
Source: BMC Bioinformatics. 2004 Oct 8;5:147. doi: 10.1186/1471-2105-5-147 (PMC528731; doi:10.1186/1471-2105-5-147)
Supplement: Additional File 2 — The original results of the above study (non-essential files are deleted to keep the file size under the limit set by BMC bioinformatics). [file 1471-2105-5-147-S2.bz2 › chilibotAdditionalFile2/dip40/379ID9927421E9792/html/NGFR_NTRK3.html]

 


 **NGFR** and **NTRK3** 
  
Found 76 abstracts in PubMed, retrieved 40.  
 

 What does Google say? 
 PDF only 
| .edu only 

---

**Interactive relationship** (e.g. stimulation, inhibition, etc)

**Inhibitory relationship**- The lack of covariation between p75NTR  [ **NGFR** ]  and trkB and trkC  [ **NTRK3** ]  regulation in aged rats indicates a changed role for p75NTR  [ **NGFR** ]  in senescent motoneurons.  Ref: 10350634 Brain Res Mol Brain Res, 1999
**Neutral relationship**- By immunohistochemistry, p75NTR  [ **NGFR** ]  alone was strongly expressed in TUNEL Bcl2 keratinocytes of the regressing outer root sheath, but both p75NTR  [ **NGFR** ]  and TrkB and or TrkC  [ **NTRK3** ]  were expressed by the nonregressing TUNEL Bcl2 secondary hair germ keratinocytes.  Ref: 11023977 FASEB J, 2000
- For nerve growth factor NGF, these are p75NTR  [ **NGFR** ]  p75 and p140trk referred to as trk or trkA, which binds both BDNF and neurotrophin NT 4 5, and trkC  [ **NTRK3** ]  receptor, which binds only NT 3.  Ref: 9106250 Neurochem Int< MedlineTA>Neurochem Int, 2000

**Non-interactive relationship** (e.g. studied together, co-existance, homology, etc.)

- This is coupled to the ectopic expression of trk B and trk C  [ **NTRK3** ] , as well as to the continued expression of trk A, and the loss of expression of p75NTR  [ **NGFR** ]  receptors.  Ref: 11027413 Prostate, 2000
- We have here examined the expression of neurotrophin receptor p75NTR  [ **NGFR** ] , trkA, trkB, and trkC  [ **NTRK3** ]  mRNA and protein in intact and axotomized primary sensory neurons of young adult 3 months and aged 30 months rats.  Ref: 10404406 J Comp Neurol, 1999
- After axotomy, a down regulation of mRNA and protein levels was observed for all neurotrophin receptors p75NTR  [ **NGFR** ] , trkA, trkB and trkC  [ **NTRK3** ]  in both young adult and aged rats.  Ref: 10404406 J Comp Neurol, 1999
- We have studied the expression of different splice forms of trkB and trkC  [ **NTRK3** ]  as well as p75  **NGFR**  in rat and mouse cochlea within this critical developmental period, using in situ hybridization, PCR, Northern blotting, and immunohistochemical analyses.  Ref: 10494076 J Comp Neurol, 1999
- Our results show that myelin formation is inhibited in the absence of functional p75NTR  [ **NGFR** ]  and enhanced by blocking TrkC  [ **NTRK3** ]  activity.  Ref: 12424382 Science, 2002
- Thus p75NTR  [ **NGFR** ]  and TrkC  [ **NTRK3** ]  receptors have opposite effects on myelination.  Ref: 12424382 Science, 2002
- We show that NT2 N cells express rRNAs for TrkA, TrkB, and TrkC tyrosine kinase  [ **NTRK3** ]  receptors and the low affinity nerve growth factor receptor  [ **NGFR** ]  p75NTR  [ **NGFR** ] .  Ref: 10386964 J Neurochem, 1999
- ... the expression of the neurotrophin, BDNF, the NT 3 receptor, TrkC  [ **NTRK3** ] , and the nonselective, low affinity neurotrophin receptor p75NTR  [ **NGFR** ] , were comparable to wild type.  Ref: 11848682 Neurobiol Dis, 2002
- Finally, an expression of trkB.T1 and trkB.T2 in oligodendrocytes, full length trkB and trkC  [ **NTRK3** ]  in nerve fibers, and p75  **NGFR**  in Schwann cells was noted at the glial interface of the VIIIth nerve during the establishment of the glial transition zone.  Ref: 10494076 J Comp Neurol, 1999
- Virtually all adult CSN express mRNA coding for the NT 3 receptor TrkC  [ **NTRK3** ]  and the BDNF receptor TrkB, and after axotomy, CSN also express mRNA for the common neurotrophin receptor p75NTR  [ **NGFR** ] , suggesting a role of endogenous neurotrophins for survival regulation of CSN.  Ref: 11236067 Rev Neurosci, 2001
- The specimens were analyzed immunohistochemically for neurotrophins nerve growth factor, brain derived neurotrophic factor, neurotrophin 3, neurotrophin 4 5 and their receptors TrkA, TrkB, TrkC  [ **NTRK3** ] , p75NTR  [ **NGFR** ] .  Ref: 11442674 Int J Urol, 2001
- Neurotrophin signaling is mediated by Trk family tyrosine kinases such as TrkA, TrkB and TrkC  [ **NTRK3** ] , as well as by the pan neurotrophin receptor p75NTR  [ **NGFR** ] .  Ref: 11836532 Nat Neurosci, 2002
- Although neurotrophins did not regulate p75NTR  [ **NGFR** ]  processing, the alpha and gamma secretase mediated cleavage of p75 is modulated by receptor tyrosine kinases Trks TrkA and TrkB but not TrkC  [ **NTRK3** ] .  Ref: 12843241 J Neurosci, 2003
- Sections of pancreatic cancer and normal pancreatic tissue were examined by immunohistochemistry for the expression of the neurotrophins NGF, BDNF, NT 3, NT 4, and their receptors TrkA, TrkB, and TrkC  [ **NTRK3** ] , as well as the low affinity receptor, p75NTR  [ **NGFR** ] .  Ref: 11561004 J Histochem Cytochem, 2001
- We have studied the expression of p75NTR  [ **NGFR** ] , trkB and trkC  [ **NTRK3** ] , respectively, in lumbar motoneurons of young adult 2 3 months and aged 30 months rats subjected to sciatic transection using in situ hybridization and immunohistochemistry.  Ref: 10350634 Brain Res Mol Brain Res, 1999
- During neuritogenesis of primary neocortical neurons, both TrkC  [ **NTRK3** ]  isoforms as well as p75NTR  [ **NGFR** ]  were located in axonal and dendritic processes.  Ref: 10998105 Eur J Neurosci, 2000
- NT 3 mediated death induction requires co signalling of TrkC  [ **NTRK3** ]  and p75NTR  [ **NGFR** ]  receptors.  Ref: 11236067 Rev Neurosci, 2001
- METHODS Immunocytochemistry and Western blot analyses were performed using primary antibodies raised against NTs nerve growth factor NGF, brain derived neurotrophic factor BDNF, NT 3, and NT 4 5 and NT receptors TrkA, TrkB, TrkC  [ **NTRK3** ] , and p75NTR  [ **NGFR** ] .  Ref: 11053307 Invest Ophthalmol Vis Sci, 2000
- Mouse PTPRO mRNA was observed in neuron populations expressing TrkA, the high affinity nerve growth factor receptor  [ **NGFR** ] , or TrkC  [ **NTRK3** ] , the neurotrophin 3 receptor  [ **NTRK3** ] , and immunoreactive mPTPRO and TrkC  [ **NTRK3** ]  colocalized in large dorsal root ganglia proprioceptive neurons.  Ref: 12532410 J Comp Neurol, 2003
- Role of neurotrophins and neurotrophins receptors in the in vitro invasion and heparanase production of human prostate cancer cells.The role of the neurotrophins and their corresponding receptors TrkA, TrkB, TrkC  [ **NTRK3** ] , and p75NTR  [ **NGFR** ]  in neoplasia has received relatively little attention.  Ref: 10545017 Clin Exp Metastasis, 1999
- With BDNF TrkB promoting survival and NT 3 TrkC  [ **NTRK3** ]  p75NTR  [ **NGFR** ]  promoting death, CSN integrate at least three different neurotrophin receptor signals for death survival decisions.  Ref: 11236067 Rev Neurosci, 2001
- A gradual shift from p75  **NGFR**  to truncated trkC  [ **NTRK3** ]  receptors in Pillar cells occurred during the formation of the tunnel of Corti.  Ref: 10494076 J Comp Neurol, 1999
- The suppressive or inhibitory influence of NT 3 and RA is the result of NT 3 binding to the low affinity receptor, p75NTR  [ **NGFR** ] , not the result of RA increasing mRNA levels for the high affinity receptor, trkC  [ **NTRK3** ] .  Ref: 10051769 Eur J Neurosci, 1999
- In order to gain insight into specific roles for TrkC  [ **NTRK3** ]  NC2 receptors during CNS neurogenesis, we compared its distribution with that of its catalytic counterparts and the p75NTR  [ **NGFR** ]  receptor in in vivo and in vitro model systems of early and late neuronal differentiation.  Ref: 10998105 Eur J Neurosci, 2000
- Spatiotemporal expression of noncatalytic TrkC  [ **NTRK3** ]  NC2 isoform during early and late CNS neurogenesis a comparative study with TrkC  [ **NTRK3** ]  catalytic and p75NTR  [ **NGFR** ]  receptors.  Ref: 10998105 Eur J Neurosci, 2000
- Differential expression of trkB.T1 and trkB.T2, truncated trkC  [ **NTRK3** ] , and p75  **NGFR**  in the cochlea prior to hearing function.  Ref: 10494076 J Comp Neurol, 1999
- Retinal degeneration upregulated both p75NTR  [ **NGFR** ]  and the high affinity neurotrophin receptor TrkC  [ **NTRK3** ]  in different parts of Müller glial cells.  Ref: 10839371 Neuron, 2000
- TrkC  [ **NTRK3** ] , the high affinity NT 3 receptor, and trkA, the high affinity NGF receptor  [ **NGFR** ] , are both expressed from the early OLP through the mature OL stage.  Ref: 8815922 J Neurosci, 1996
- We have used immunofluorescence techniques with a panel of antibodies against known neurotrophin receptors trk A, trk B, trk C  [ **NTRK3** ] , p75NTR  [ **NGFR** ]  to map the locations of these receptors in the developing neuromuscular system of mice from our neurotrophin 3 NT 3 knockout colony.  Ref: 12141445 Int J Dev Biol, 2002
